# A New Perspective on the Multidimensionality of Divergent Thinking Tasks

**DOI:** 10.3389/fpsyg.2019.00985

**Published:** 2019-05-03

**Authors:** Boris Forthmann, Paul-Christian Bürkner, Carsten Szardenings, Mathias Benedek, Heinz Holling

**Affiliations:** ^1^Institut für Psychologie, University of Münster, Münster, Germany; ^2^Fakultät Statistik, TU Dortmund University, Dortmund, Germany; ^3^Institut für Psychologie, University of Graz, Graz, Austria

**Keywords:** IRTrees, item-response theory, dimensionality, creativity, creative quality, fluency, divergent thinking

## Abstract

In the presented work, a shift of perspective with respect to the dimensionality of divergent thinking (DT) tasks is introduced moving from the question of multidimensionality across DT scores (i.e., fluency, flexibility, or originality) to the question of multidimensionality within one holistic score of DT performance (i.e., snapshot ratings of creative quality). We apply IRTree models to test whether unidimensionality assumptions hold in different task instructions for snapshot scoring of DT tests across Likert-scale points and varying levels of fluency. It was found that evidence for unidimensionality across scale points was stronger with be-creative instructions as compared to be-fluent instructions which suggests better psychometric quality of ratings when be-creative instructions are used. In addition, creative quality latent variables pertaining to low-fluency and high-fluency ideational pools shared around 50% of variance which suggests both strong overlap, and evidence for differentiation. The presented approach allows to further examine the psychometric quality of subjective ratings and to examine new questions with respect to within-item multidimensionality in DT.

## Introduction

Divergent thinking (DT) tasks are one of the most important proxies of creative thinking (Runco and Acar, 2012). For example, they are frequently used in research on the link of intelligence and creativity (e.g., Karwowski et al., 2016) and have been shown to predict creative achievement above intelligence (Kim, 2008). These tasks typically ask participants to come up with either many or creative ideas in order to solve a given problem. A classic research issue relating to such tasks is their underlying dimensionality; this has been discussed and researched since the 1950’s. Most of the studies in this vein addressed the factorial validity of Guilford’s classic four divergent-thinking abilities (i.e., fluency, flexibility, elaboration, and originality) in several common tests and batteries (e.g., Hargreaves and Bolton, 1972; Follman et al., 1973; Plass et al., 1974; Aliotti et al., 1975; Heausler and Thompson, 1988).

In the current work, however, we focus on the dimensionality of creative quality. In line with Forthmann et al. (2017a), we refer to creative quality scores as all scorings that have a clear definitional link to the concept of creativity. Prominently, originality scores fit this notion of creative quality well, because originality is one of the defining characteristics of creativity (e.g., Runco and Jaeger, 2012). Thus, all commonly used indicators of originality, such as uncommonness, remoteness, and cleverness (see Wilson et al., 1953), are examples of creative quality scores (see Forthmann et al., 2017a). Moreover, this definition of creative quality allows including usefulness-based scores as it is the second defining characteristic of creativity (e.g., Runco and Jaeger, 2012). Beyond the two-componential standard definition of creativity, the definition of creative quality is open to other conceptualizations of creativity such as Simonton’s three-componential definition (Simonton, 2012, 2018) or Kharkhurin’s four-criterion definition (Kharkhurin, 2014), for example.

Various examples of quality scores can be found in the literature on DT. First, originality has been scored with respect to uncommonness (e.g., Runco and Charles, 1993; Mouchiroud and Lubart, 2001), remoteness (e.g., Christensen et al., 1957; Piers et al., 1960; Parnes, 1961), and cleverness (e.g., French et al., 1963; Mullins, 1963; Forthmann et al., 2017a). Second, usefulness has been scored independent of originality (e.g., Runco and Charles, 1993; Diedrich et al., 2015; Olteteanu and Falomir, 2016). Third, both components of the standard definition of creativity – originality and usefulness – were combined in an all-in-all creativity score (e.g., Runco and Mraz, 1992; Runco and Charles, 1993; Mouchiroud and Lubart, 2001; Storm and Patel, 2014; Diedrich et al., 2015). Clearly, there is no unanimous way to score DT responses for creative quality (see also Reiter-Palmon et al., 2019). In the current work, a creative quality score that is based on all classic indicators of originality will be used because this approach has been used more extensively over recent years (e.g., Silvia et al., 2008, 2009; Hass, 2015, 2017; Hofelich Mohr et al., 2016).

Subjective ratings with Likert-scales are often used to score creative quality (e.g., Silvia et al., 2008). In particular, providing subjective ratings for a person’s ideational pool (all generated responses as a whole; see description below) will be in the focus of the current work (Runco and Mraz, 1992; Silvia et al., 2009). In fact, these ratings raise new questions with respect to dimensionality issues of DT tests. By a combination of ratings and a particular item-response modeling technique, so called IRTrees (De Boeck and Partchev, 2012), we show how to assess whether providing few or many ideas might induce multidimensionality in rated creative quality. Is there a relationship of quality in low-fluency sets and quality in high-fluency sets within the same person? Clearly, for the measurement of quality it is fundamental to know if the ability to be creative is being measured no matter how many ideas are generated. In fact, it is discussable if high-fluency and low-fluency ideational pools are unidimensional with respect to creative quality. Strategies that are efficient in terms of fluency (such as retrieval from memory) are most likely not conducive to idea quality which requires more demanding strategies (Gilhooly et al., 2007). Thus, differences in cognitive processes when people are fluent and when they are not may affect the dimensionality of creative quality scores.

In a similar vein, studies on the “be-creative” effect in DT are informative (Christensen et al., 1957; Harrington, 1975; Runco, 1986; Runco et al., 2005; Nusbaum et al., 2014; for be-creative effects in other creative performance tasks see, for example, Chen et al., 2005; Niu and Liu, 2009; Rosen et al., 2017). In this line of research, standard instructions (be-fluent instructions) with a focus on quantity of responses (*think of as many ideas as possible*) are compared with explicit instructions to be creative (be-creative instructions; *think of ideas that are creative, original, uncommon, clever, and so forth*). Substantial variation in participants’ strategies is expected, when the task goal remains opaque as in be-fluent instructions (Harrington, 1975; Nusbaum et al., 2014). On the contrary, “be-creative” instructions are assumed to homogenize participants’ mindset toward the task and more demanding strategies can be expected to be used from the beginning (Nusbaum et al., 2014). Guilford (1968) argued also that explicit instructions to generate rather creative responses are likely to change the cognitive processes during idea generation. He expected a stronger involvement of evaluative processing with explicit instructions, which is in line with recent work by Nusbaum et al. (2014). As a consequence, the involvement of evaluative processing should be more homogeneous across participants when receiving explicit instructions to be creative as compared to be-fluent instructions. Thus, multidimensionality of creative quality of low-fluency and high-fluency ideational pools seem to be more likely under be-fluent instructions.

Moreover, the introduced modeling technique allows addressing another issue regarding the dimensionality of DT quality. This issue is related to the subjective scoring of DT quality. Subjective scorings of DT have gained prominence over the last years, but they were used also in the early years of DT research. One example of subjective scoring is the so-called snapshot scoring or scoring of ideational pools (Runco and Mraz, 1992; Silvia et al., 2009). Here raters see a full ideational pool of a participant and give a rating of the set’s overall creative quality. These ratings are typically given on a 5-point Likert scale and here the question can be asked: Do we measure the same quality at the lower end and the upper end of the scale? Or to put it differently: Is one latent trait underlying the full rating scale? This question has been coined *ordinal hypothesis* by De Boeck and Partchev (2012).

### An IRTree Modeling Approach for Divergent-Thinking Creative Quality Ratings

**FIGURE 1:**
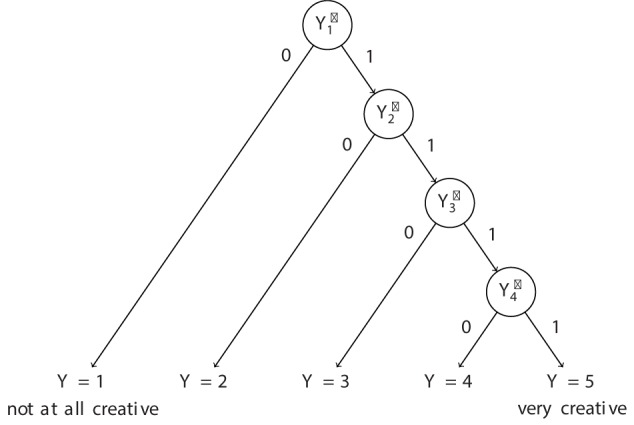
Linear response tree for the creative quality ratings. The $Y_{r}^{*}$ represent sub-items (De Boeck and Partchev, 2012) and the Y the response categories for the ratings.

The model we use here is an IRT model for rating scales. It is inspired by the sequential model of Tutz (1990). In order to understand the model, it is helpful to use a linear response tree structure of the Likert scale (see Figure 1). A linear response tree is comprised of end nodes *Y* (i.e., the concrete response categories of the Likert scale) and internal nodes *Y*^∗^ (De Boeck and Partchev, 2012). As shown in Figure 1, internal nodes can be understood as binary-coded sub-items. For example, the sub-item for the first internal node ($Y_{1}^{*}$ in Figure 1) in the tree is coded zero when an ideational pool receives a rating of one, and the sub-item is coded one when the ideational pool received a rating larger than one. The sub-item for the second internal node ($Y_{2}^{*}$ in Figure 1) in the tree is coded zero when an ideational pool receives a rating of two, and the sub-item is coded one when the ideational pool received a rating larger than two, and so forth. Hence, the ratings on the Likert scale are divided into binary-coded sub-items $Y_{pir}^{*}$ for each of the *r*=1,…, *R* internal nodes. Please note that for simplicity we will use the term node from here onward to only refer to internal nodes of a response tree. Each node in the linear tree represents a branch with probability to remain at a given category *m* = 1,…,*M* - 1 in contrast to all possible higher categories (De Boeck and Partchev, 2012). The model is based on the sub-items and can be formulated as:

$$P(Y_{pir}^{*}=y_{pir}^{*}|\theta_{p})=\exp(\theta_{pr}+\beta_{ir})/[(1+\exp(\theta_{pr}+\beta_{ir}))],$$

with θ_*p*_ = (θ_*p*1_,…,θ_*pr*_) being the node-specific latent variables (with person number *p* = 1,…,*P*) and β_*ir*_ (with item number *i* = 1,…,*I*) denoting a node-specific item threshold. For unidimensional models, only one latent ability is assumed to underlie the linear response tree. We will also use the term *node-unidimensionality* to refer to such unidimensional models. Moreover, as outlined in De Boeck and Partchev (2012), such a linear response tree can be combined with latent variable trees, which leads to other multidimensional modeling options. For example, a bi-factor structure can be proposed and tested as an underlying structure of a linear response tree.

### Aim of the Current Study

The aim of the current study is to provide new insights into the dimensionality of DT by means of a proof of concept study using IRTree modeling. The IRTree modeling framework allows assessing dimensionality issues that are at the heart of frequently used scoring procedures in divergent-thinking assessment such as snapshot scoring of ideational pools (Runco and Mraz, 1992; Silvia et al., 2009). Related to this scoring procedure, we show how to test a unidimensional model underlying the rating scale against a multidimensional alternative that includes a different latent variable at each of the respective nodes. Unidimensionality of ratings in snapshot scoring with respect to the used scale is in fact fundamental to the method, but it has not yet been assessed in the literature. It is important to know if the ability to score better as the lowest scale point is the same ability that allows a person to move from the second highest to the highest scale point of the response tree.

Moreover, by means of IRTree modeling, it is possible to take a different view on the relationship between fluency and creative quality ratings. We can assess if the latent dimensions underlying low-fluent and high-fluent creative quality are related or even tap to the same latent variable. This question was tested for standard and be-creative instructions. The goal of this research is to illustrate a methodological approach to new dimensionality issues of DT which moves from the question of multidimensionality across DT scores to the question of multidimensionality across the scale of DT scores and across ideational pools of varying size.

## Materials and Methods

### Participants

The data set to illustrate the above ideas was taken from Forthmann et al. (2016). This dataset was gathered for a large project and used in previous publications on DT assessment issues (Forthmann et al., 2016, 2017a,b) and the relationship between multicultural experiences and DT performance (Forthmann et al., 2018). However, the analyses in this study are unique to this work and go beyond any of the issues tackled in the above-mentioned articles. In that study eight Alternate Uses Tasks (AUTs; Wallach and Kogan, 1965; Guilford, 1967) were administered online. Four of the tasks were administered with a be-fluent instruction and the other four with a be-creative instruction. The AUTs were scored for fluency and subjective ratings of creative quality of the full ideational pools were given by two raters. The raters were asked to give holistic ratings of creative quality reflecting the three classic originality indicators – uncommonness, remoteness, and cleverness – according to the rating scheme provided in Silvia et al. (2008): *Uncommonness: Any response that is given by a lot of people is common, by definition*; *remoteness: creative uses for an object are far from everyday uses and obvious responses*; and *cleverness: clever ideas strike people as insightful, ironic, humorous, fitting, or smart*. In addition, the coding scheme refers also to task appropriateness: *A random or inappropriate response would be uncommon but not creative*. For more details with respect to these scoring dimensions see Silvia et al. (2008; p. 85). The ratings were provided on a 5-point Likert-scale as depicted in Figure 1. Absolute agreement intra-class correlations for the average scores indicated good inter-rater reliability according to Cicchetti’s criteria (Cicchetti, 2001), ICC(2,2) = 0.701, 95%-CI: [0.669, 0.743]. The final sample for data analysis was *N* = 249 (age: *M* = 23.48, *SD* = 6.44; gender: 79.12% female and 20.88% male). For more details please consult Forthmann et al. (2016).

This study was carried out in accordance with the recommendations for online studies provided by the ethics committee of the department of psychology of the University in Münster. These guidelines are in accordance with the guidelines provided by the German foundation for online research (Deutsche Gesellschaft für Online Forschung). All subjects gave written informed consent prior to participation. An ethics approval was not required as per institutional and national guidelines.

### Data Preparation and Statistical Analysis

The data were prepared with R functions provided by the IRTrees package (De Boeck and Partchev, 2012). In order to fit the proposed models, the function dendrify() was used to expand and reshape the data from a wide-format matrix into a long-format matrix of sub-item responses (see De Boeck and Partchev, 2012). The data frame that is built by this function includes an indicator variable for the nodes, so that unidimensionality vs. multidimensionality for the nodes can directly be tested.

Analogous to Partchev and De Boeck (2012) median splits were used in order to separate low-fluency ideational pools from high-fluency ideational pools. Moreover, a methodological sensitivity analysis was performed to assess the differences between median-splits (with respect to fluency) within items and within persons. For the within-item median split all medians for every AUT task were calculated separately for both instructions. An indicator variable for low-fluency vs. high-fluency ideational pools was then formed by a binary coding. The indicator was coded one if fluency for a given set was strictly below (instead of below or equals) the respective median since this led to a more balanced distribution of the indicator variable. The within-person split was analogous, but here the median values were calculated for each person and both instruction types separately. Based on these medians the same indicator variables were built. Moreover, possible differences between raters’ central tendency of ratings were statistically controlled by including a corresponding fixed effect in all estimated models.

De Boeck and Partchev (2012) used maximum-likelihood methods to fit their models. For the present paper, however, we prefer a Bayesian approach for two reasons: First, random effects in the models refer to latent traits and latent trait correlations are of particular interest for our research question. Typical fitting procedures only return a point estimate for these correlations. Bayesian methods return the full posterior distribution of the correlations including standard errors and credible intervals (i.e., Bayesian confidence intervals). These provide a better understanding of the correlation structure and facilitate its interpretation. This is particularly relevant, if the posterior distribution of correlations is skewed, in which case the maximum-likelihood estimate is a poor measure of central tendency. Second, Bayesian methods have some advantages when it comes to model comparison. When using maximum-likelihood procedures, model comparisons are usually performed either by means of significance tests or by comparing information criteria. The former often lack statistical power when the number of observations is small, but for large data sets, even tiny and practically irrelevant differences in model fit will often get significant. These drawbacks are partially addressed by information criteria such as the AIC, but it remains unclear how much the criteria of two models should differ to indicate substantial differences in model fit. For recently developed Bayesian specific information criteria, namely an approximation of the leave-one-out cross-validation (LOO), standard errors can be computed giving a much better sense of uncertainty in those criteria (Vehtari et al., 2017).

All statistical analyses were performed in R (R Core Team, 2013) using the brms package (Bürkner, 2016), which is based upon the probabilistic programming language Stan (Carpenter et al., 2017). It is further noteworthy, that refitting the models by means of the package lme4 (Bates et al., 2015) which uses restricted maximum likelihood estimation did not lead to relevant differences of the presented findings. The R code and data file are available in Open Science Framework^1^.

## Results

### Dimensionality Underlying the Nodes

A unidimensional linear-response tree model was compared with a model that assumed different latent variables for each of the corresponding nodes. This comparison was initially conducted for the full data set (thus, instruction as an influencing variable was ignored). This comparison was in favor of the multidimensional model as indicated by the difference in LOO criteria (see Table 1). When inspecting the correlation matrix of this multidimensional model it was apparent that latent variables referring to nodes of close proximity were more strongly correlated as compared to distant nodes. The three highest nodes correlated fairly strong with each other, whereas the lowest node correlated moderately (*r* = 0.39) with its next node and appeared to be unrelated to the other nodes. Thus, the ability to depart from the most common ideas (the ability to generate a response that receives a rating >1) appeared to be loosely related to the other abilities underlying the response tree. However, this pattern of results might be a reflection of different underlying strategies that either opt for quantity with the be-fluent instruction – participants are likely to accept low-quality ideas to increase fluency more readily here – or for quality with the be-creative instruction. Consequently, the dimensionality underlying the nodes was tested again for both instruction types separately.

**Table 1:** Comparison of unidimensional models vs. multidimensional models with respect to the nodes for the full model and for the be-creative instruction and be-fluent instruction.

	Node 1	Node 2	Node 3
**Full model**			
Node 2	0.38 [0.19, 0.57]	–	
Node 3	0.02 [-0.21, 0.26]	0.79 [0.65, 0.91]	–
Node 4	-0.13 [-0.50, -0.23]	0.56 [0.23, 0.81]	0.85 [0.59, 0.98]
**Be-fluent**			
Node 2	0.61 [0.37, 0.83]	–	
Node 3	0.35 [-0.11, 0.76]	0.77 [0.51, 0.96]	–
Node 4	NA	NA	NA
**Be-creative**			
Node 2	NA	–	
Node 3	NA	0.89 [0.74, 0.98]	–
Node 4	NA	0.73 [0.38, 0.96]	0.80 [0.52, 0.98]

	**LOO (*SE*)**		**ΔLOO (*SE*)**

**Full model**			
Unidimensional	8618.21 (113.09)		
Multidimensional	8397.68 (110.45)		220.52 (30.10)
**Be-fluent**			
Unidimensional	3742.83 (77.52)		
Multidimensional	3711.31 (75.90)		31.51 (13.23)
**Be-creative**			
Unidimensional	3882.10 (66.76)		
Multidimensional	3889.99 (67.33)		-7.89 (6.39)


*n = 249. The unidimensional model implies the same latent variable for all nodes. LOO, information criterion approximating leave-one-out cross-validation; ΔLOO, LOO-difference between multidimensional and unidimensional models; NA, information in the data was insufficient for the highest node in the be-fluent instruction and the lowest node in the be-creative instruction.*

For the instruction-specific tests of node-specific multidimensionality the item-parameters for the fourth node in the be-fluent instruction and the first node in the be-creative instruction were not identified due to lack of idea sets that received such ratings. According to the LOO information criterion, the multidimensional node model fit better in the be-fluent instruction, whereas for the be-creative instruction the unidimensional model was better (see Table 1). From the correlation estimates for latent variables of these models it is evident that support for unidimensionality of the nodes was stronger with a be-creative instruction as compared to the be-fluent instruction. The inter-correlations of latent variables underlying node 1, node 2, and node 3 ranged from *r* = 0.37 to *r* = 0.76 in the be-fluent instruction, whereas for the be-creative instruction the range was *r* = 0.73 to *r* = 0.89.

### Discriminating Creative Quality for Ideational Pools of High and Low Fluency

In order to account for insufficient information in the data at the first node in the be-creative instruction and the fourth node in the be-fluent instruction, those were again omitted. Then, separate analyses for both instructions were conducted (however, the interested reader will find results for the full dataset in the supplementary material or in the OSF see text footnote 1). Initially, the competing models were compared by means of the LOO criterion. With respect to the median splitting method, it was found that using within-person split indicators for high-fluency and low-fluency ideational pools performed better in terms of model fit as compared to the unidimensional model, and the within-item split indicators. Generally, modeling two latent variables for high vs. low-fluency sets increased model fit as compared to a unidimensional model (see Tables 2, 3).

**Table 2:** Correlations of latent variables and model comparison results for a differentiation of low-fluency and high-fluency creative quality for be-fluent instructions only.

	High-fluency
**Within-person split**	
Low-fluency	0.69 (0.54, 0.83)
**Within-item split**	
Low-fluency	0.63 (0.33, 0.89)

	**LOO (*SE*)**

**Model**	
Unidimensional	3742.83 (77.52)
**Within-person split**	
Low-fluency vs. high-fluency	3693.82 (77.21)
ΔLOO (vs. unidimensional)	49.01 (15.50)
**Within-item split**	
Low-fluency vs. high-fluency	3725.43 (77.48)
ΔLOO (vs. unidimensional)	17.39 (10.32)


*LOO, information criterion approximating leave-one-out cross-validation; ΔLOO, LOO-difference between multidimensional and unidimensional models.*

**Table 3:** Correlations of latent variables and model comparison results for a differentiation of low-fluency and high-fluency creative quality for be-creative instructions only.

	High-fluency
**Within-person split**	
Low-fluency	0.68 [0.53, 0.81]
**Within-item split**	
Low-fluency	0.72 [0.50, 0.90]

	**LOO (*SE*)**

**Model**	
Unidimensional	3882.10 (66.76)
**Within-person split**	
Low-fluency vs. high-fluency	3827.73 (67.30)
ΔLOO (vs. unidimensional)	54.37 (14.98)
**Within-item split**	
Low-fluency vs. high-fluency	3859.44 (66.98)
ΔLOO (vs. unidimensional)	22.66 (8.88)


*LOO, information criterion approximating leave-one-out cross-validation; ΔLOO, LOO-difference between multidimensional and unidimensional models.*

We then inspected the pattern of correlations and found estimates for within-item split of *r* = 0.66 (be-fluent; see Table 2) and *r* = 0.73 (be-creative; see Table 3) which implies that at least 44% of variation can be considered as common. Similarly, for the within-person split correlations of *r* = 0.71 (be-fluent; see Table 2) and *r* = 0.70 (be-creative; see Table 3) were found which implies an amount of shared variation of 49%. Thus, besides a strong construct overlap of creative quality for low-fluency and high-fluency ideational pools, there is also evidence for differentiation at the latent level. Moreover, the strong overlap of credible intervals for each of the correlations should be noted which indicates that the found slight differences between correlation estimates are negligible. Consequently, construct differentiation of low-fluency and high-fluency ideational pools with respect to creative quality latent variables can be considered to be similar for both instructions and both splitting methods (within-person or within-item).

## Discussion

The dimensionality of divergent-thinking indices such as originality and fluency has been an intriguing endeavor since the early years of creativity research. In the current study, we introduced IRTrees as an interesting model alternative to study the underlying dimensionality of DT. For example, we assessed the dimensionality of creative quality ratings over the rating scale in order to test the ordinal hypothesis of ratings (De Boeck and Partchev, 2012). In addition, within the IRTree approach, we shifted the focus from the relationship of quality and quantity and the corresponding debate on the lack of discriminant validity of fluency and originality toward a quality-quality relationship when creative quality is assessed for high-production and low-production ideational pools within two common important instructions. Altogether, this study represents a new perspective on the dimensionality of divergent-thinking scores away from classical factor analytic approaches.

Using subjective ratings is not free of criticism (see Runco, 2008) and testing the null hypothesis of node unidimensionality offers a way to empirically test how well they work and under which circumstances. For example, these findings highlight recent suggestions made by Reiter-Palmon et al. (2019) to carefully take instruction-scoring fit into account. Instruction-scoring fit refers to the congruence of instructions and scoring, for example, a be-creative instruction resonates well with a scoring of creative quality. While instruction-scoring fit is already preferable from a conceptual perspective, the findings of the current study highlight the importance of instruction-scoring fit because node-unidimensionality as a desirable psychometric property of subjective ratings was better supported when instructions and scoring were congruent. Thus, a Likert-type scale seems to work only in the intended way here if raters see responses of participants who were instructed to focus on quality rather than quantity. Most likely, this finding can be attributed to more consistent behavior as a consequence of a clear goal that is set by means of the instructions (e.g., Harrington, 1975; Nusbaum et al., 2014). Thus, this work presents a starting point to further investigate if unidimensionality underlying the rating scale can be maintained only for be-creative instructions, which should be tested in other samples (perhaps relying on a larger rater sample) in order to corroborate the observations here.

Tentatively, we conclude that subjective ratings of creative quality require instruction-scoring fit to be psychometrically sound. However, instruction-scoring fit should not be viewed as a dichotomy. For example, the original test material of the AUT from the Guilford group (Wilson et al., 1960) instructs participants to think of uses that are different from an object’s most common use (which is also provided for each object during the test procedure). Thus, instruction-scoring fit for creative quality and the original Guilford instruction would be slightly higher as compared to the fully unrestricted be-fluent instruction used in the current study. Instruction-scoring fit could even be further enhanced when instructions to avoid the most common use (Wilson et al., 1960) are further supplemented by commonly generated example ideas which are also required to be avoided (Shin et al., 2018; George and Wiley, 2019). Hence, it is yet unclear which degree of instruction-scoring fit (with explicit instructions to be creative representing maximum instruction-scoring fit when creative quality is of interest) is required for subjective ratings to be node-unidimensional.

In addition, with be-fluent instructions it is efficient to quickly retrieve as many ideas from memory as possible (similar to verbal fluency tasks; for a related discussion see Nusbaum et al., 2014). Idea sets resulting from such a strategy reflect production ability and are likely to receive rather low ratings on a 5-point scale. Otherwise some participants may still receive higher-ratings even when be-fluent instructions are given due to their need to be original, need for cognition, or other motivational state and in order to reach such higher ratings more demanding strategies are necessary. Consequently, such differences in underlying cognitive processes and strategies to generate idea sets of varying quality may have caused the observed lack of node-unidimensionality. In fact, from experimental psychology it is known that differences in cognitive processes may influence the dimensionality of latent variable models (Oberauer et al., 2005).

For measurement of creative quality, a unidimensional model that outperforms multidimensional alternatives is desirable. This seems to be particularly the case when participants receive a be-creative instruction. If participants look for imaginative ideas und do not focus on quantity, fluency can be conceived as a byproduct and differentiating for fluency should not affect unidimensionality of creative quality. However, it was illustrated that high-fluency and low-fluency ideational pools differ in terms of creative quality at the latent level (around 50% of the variance is shared). But how did this heterogeneity of latent traits emerge?

One potential explanation here is to assume that differential effects of practice, exhaustion, and current motivation affected the dimensionality here. First, it has been demonstrated that at late time points in DT different and more elaborate strategies are more likely to be used (Gilhooly et al., 2007; see also below). Thus, if eight tasks are administered such strategies can directly be used in later tasks if they are encountered/used while working on one of the previous tasks. Second, if participants get more and more exhausted over the course of testing, it is likely that cognitive resources are used in different ways. Similarly, Haager et al. (2016) showed that production tasks are perceived to be less and less interesting over the course of time, while at the same time performance drops. As a consequence, motivational states toward the end of a test session with multiple DT tasks are likely to be less beneficial for performance. Most likely, all of the above factors have affected dimensionality in the current sample. Furthermore, early positions of the objects during test administration in the current sample were more likely to result in fluency scores above the median and, consequently, indicators for high-fluency and low-fluency latent variables were unevenly coded across their position during administration.

Another explanation for the differentiation of creative quality between low-fluency and high-fluency ideational pools could be that raters judged the quality of pools with varying numbers of responses in a slightly different way. In fact, it has been demonstrated that the amount of information that needs to be judged has a detrimental effect on rater agreement when DT responses are rated for creative quality (Forthmann et al., 2017b). Thus, it should not be overlooked that processing on the side of the raters might potentially influence the results here beyond the statistical control of rater severity effects as it was applied in the current study.

The differentiation between low-fluency and high-fluency ideational pools (reflecting differences in speed of the response generation) bears further interesting opportunities for the study of within-item multidimensionality. Ratings can be obtained for individual responses generated for the same item, and responses could also be grouped according to certain characteristics that can be assumed to influence within-item dimensionality. For example, a well-known phenomenon is the serial order effect (for example, Beaty and Silvia, 2012) in DT. It describes the tendency to generate ideas of better quality (for example, more original and remote ideas) toward the end of the allotted time on task. This effect points to differences in the underlying cognitive processes at the beginning and at the end of a test session. Consequently, it might be suggested that different abilities are involved over the time course and this hypothesis can be tested by the method outlined here.

Finally, it needs to be acknowledged that the current study is indeed limited to the creative quality scoring that was used, namely a holistic scoring of uncommonness, remoteness, and cleverness. Forthmann et al. (2017a) found strong correlations between such holistic ratings and ratings of the cleverness dimension only (*r* = 0.82 for latent variables), it is likely that subjective ratings for the cleverness dimension yield similar results as compared to the current work. It is, however, unclear if results generalize to other originality indicators, indicators of the usefulness component of the standard definition of creativity, or other indicators relating to components specific to alternative conceptions of creativity (e.g., Simonton, 2012, 2018; Kharkhurin, 2014). In relation to this, it should further be mentioned that a test of node-unidimensionality is a specific issue for subjective ratings relying on Likert-type scales, whereas the question of multidimensionality of creative quality as a function of the ideational pool size (low-fluency vs. high-fluency) might also be addressable for other ways to score creative quality (e.g., statistical frequency as an indicator of originality). In this regards, future studies are indeed needed to further expand our knowledge.

### How to Detect and Deal With Technical Problems

In order to apply IRTrees in future studies, our work provides guidance on how to deal with possible technical problems such as not enough information at some of the nodes. Here it was demonstrated that the available information strongly depended on the instruction. That is, not enough information was available for the fourth node with a be-fluent instruction and for the first node with a be-creative instruction. As a consequence, all available data for those nodes in instruction-specific models were excluded. In future attempts, the available information at all nodes should be initially checked. For example, it normally occurs that standard errors for fixed effects of non-informative nodes are unrealistically high. Furthermore, in Bayesian applications non-informative nodes can be detected by convergence problems and high correspondence of prior and posterior if proper priors are assigned to the respective parameters.

## Overall Conclusion

In the current work, IRTrees were introduced as a method that allows a change of perspective on the multidimensionality of DT tasks. The methodology looks promising in order to further explore the psychometric quality of subjective ratings of DT ideational pools (which has been traditionally a hot topic in DT research). Moreover, new studies on within-item multidimensionality of DT and also multidimensionality due to practice, exhaustion, and motivation are promising future applications of the presented method.

## Ethics Statement

This study was carried out in accordance with the recommendations for online studies provided by the institutional ethics committee. These guidelines are in accordance with the guidelines provided by the German foundation for online research (Deutsche Gesellschaft für Online Forschung). All subjects gave informed consent prior to participation. An ethics approval was not required as per institutional and national guidelines.

## Author Contributions

BF designed the study, wrote the initial draft, analyzed the data, and revised the manuscript. P-CB analyzed the data and revised the manuscript. CS wrote the initial draft and revised the manuscript. MB revised the manuscript. HH designed the study and revised the manuscript.

## Conflict of Interest Statement

The authors declare that the research was conducted in the absence of any commercial or financial relationships that could be construed as a potential conflict of interest.

## Footnotes

1 https://osf.io/3rf24/
